# Extracellular Sphingosine-1-Phosphate Downstream of EGFR Increases Human Glioblastoma Cell Survival

**DOI:** 10.3390/ijms22136824

**Published:** 2021-06-25

**Authors:** Rosaria Bassi, Stefania Brambilla, Cristina Tringali, Paola Giussani

**Affiliations:** Department of Medical Biotechnology and Translational Medicine, Università degli Studi di Milano, LITA Segrate, Via Fratelli Cervi, 93, 20090 Segrate, Italy; rosaria.bassi@unimi.it (R.B.); stefania.brambilla2@studenti.unimi.it (S.B.); cristina.tringali@unimi.it (C.T.)

**Keywords:** epidermal growth factor receptor, cell survival, sphingosine kinase 1, sphingosine-1-phosphate

## Abstract

Sphingosine-1-phosphate (S1P) is a crucial mediator involved in the progression of different cancers, including glioblastoma multiforme (GBM), the most frequent and deadly human brain tumor, characterized by extensive invasiveness and rapid cell growth. Most of GBMs overexpress the epidermal growth factor receptor (EGFR), and we investigated the possible link between S1P and EGFR signaling pathways, focusing on its role in GBM survival, using the U87MG human cell line overexpressing EGFR (EGFR+). We previously demonstrated that EGFR+ cells have higher levels of extracellular S1P and increased sphingosine kinase-1 (SK1) activity than empty vector expressing cells. Notably, we demonstrated that EGFR+ cells are resistant to temozolomide (TMZ), the standard chemotherapeutic drug in GBM treatment, and the inhibition of SK1 or S1P receptors made EGFR+ cells sensitive to TMZ; moreover, exogenous S1P reverted this effect, thus involving extracellular S1P as a survival signal in TMZ resistance in GBM cells. In addition, both PI3K/AKT and MAPK inhibitors markedly reduced cell survival, suggesting that the enhanced resistance to TMZ of EGFR+ cells is dependent on the increased S1P secretion, downstream of the EGFR-ERK-SK1-S1P pathway. Altogether, our study provides evidence of a functional link between S1P and EGFR signaling pathways enhancing the survival properties of GBM cells.

## 1. Introduction

Glioblastoma multiforme (GBM) is the most frequent and aggressive primary tumor affecting the central nervous system in humans. GBMs are characterized by high invasive proliferation and resistance to current therapeutic intervention. These characteristics lead to one of the worst survival rates of all the human cancers [1,2]. Like most aggressive cancers, GBMs are characterized by distinct molecular and genetic alterations leading, among different properties, to malignant growth [3]. Among the most frequent alterations occurring in GBM, mutations and/or overexpression of several growth factor receptors have been observed. In addition, the signals transduction pathways (such as p53, phosphatidylinositol-3 kinase/Akt, and Ras/MEK/ERK) downstream these receptors are altered, leading to aberrant proliferation and invasiveness of GBM cells [4]. Over 50% of GBM patients present a very high rate of epidermal growth factor receptor (EGFR) mutations [5]; in particular, the oncogene epidermal growth factor receptor variant III (EGFRvIII), a constitutively active mutant form of the receptor, is among the most common EGFR mutations in GBM. The presence of this EGFR variant has been associated with increased tumor proliferation even if its functional and biological significance in GBM has not yet been completely clarified in terms of its relationship with other molecular alterations observed in these tumors. An increasing number of papers demonstrate that sphingolipids, in particular sphingosine-1-phosphate (S1P), are involved in the control of cell fate in physiological as well as in pathological conditions [3,6,7,8]. It is widely known that S1P has a key role in the regulation of different aspects of cancer pathogenesis and therapeutics [9,10,11,12,13,14,15,16]. S1P is produced from sphingosine and ATP by two isoforms of the enzyme sphingosine kinase (sphingosine kinase 1 (SK1) and 2 (SK2)) [17]. S1P, in turn, can be dephosphorylated back to sphingosine or can be irreversibly cleaved to hexadecenal and phosphoethanolamine [18]. S1P released by cells in the extracellular milieu can act as an autocrine as well as a paracrine factor interacting with five specific receptors (S1P_1–5_) coupled to G-protein, activating several signal transduction pathways [19,20,21]. S1P is recognized as an onco-promoter molecule in many tumors, including GBM [12,22]. There are several studies demonstrating that S1P can promote the malignant behavior of GBM cells by enhancing their proliferation, motility and invasiveness [23,24,25]. Most of the oncogenic effects exerted by S1P are related to its increased production via SK1. In fact, SK1 levels are elevated in GBM cell lines [26,27], and the inhibition of this enzyme (by chemical inhibitors as well as by siRNA) reduces xenografts and human GBM cell growth [28]. Furthermore, the expression of SK1 in GBM is inversely correlated with patient survival [16].

It is known from the literature that S1P regulates EGFR expression in cancer as well in non-cancer cells [29,30], and we recently demonstrated the existence of a cross-talk between S1P and EGFR signaling pathways in the regulation of human GBM cell invasiveness [31].

To better elucidate the functional interplay between S1P and EGFR signaling pathways in human GBM cell growth, we analyzed the survival properties of two human GBM cell lines differing only for the expression of EGFRvIII in different experimental conditions. The results of the present study indicate that extracellular S1P acts via S1P_1_ downstream of EGFR as a fundamental pro-survival signal in GBM cells.

## 2. Results

### 2.1. EGFRvIII Expression Correlates with Higher Survival Rate in U87MG Cells

In addition to supporting invasiveness, EGFR signaling is known to promote cell proliferation and survival [32]. Therefore, we evaluated the influence of EGFR expression on GBM survival by comparing the survival rate of two human GBM cell lines, the EGFR- and EGFR+ cells after TMZ treatment. To this purpose, increasing concentrations of TMZ (ranging from 100 to 200 μM) were used to treat EGFR- and EGFR+ cells, and the cytotoxic effect on cells was evaluated by the analysis of cell viability by MTT assay.

**Figure 1:**
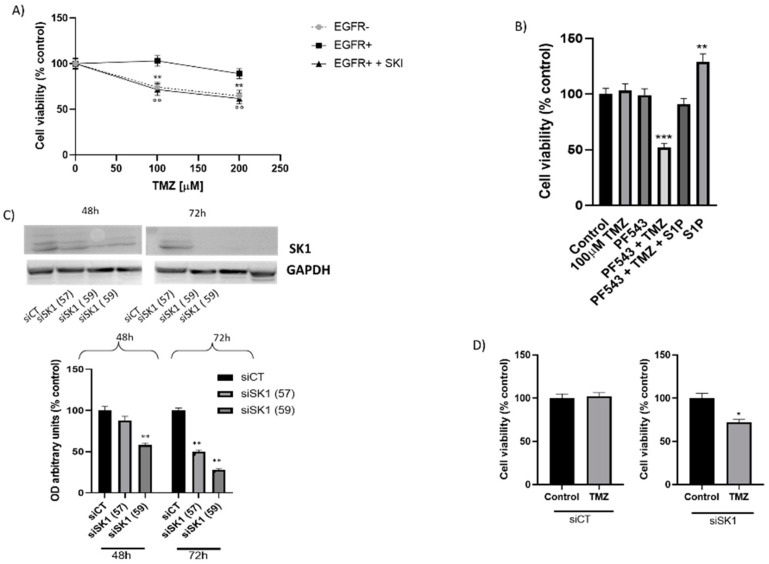
**Role of S1P-SK1 on survival properties of EGFR+ cells.** (**A**) EGFR- (●) and EGFR+ cells were seeded at 20,000 and 30,000 cells/cm^2^ exposed to different doses of TMZ (100–200 µM) alone (■) or in combination with 4 µM SKI (▲). Cell viability was assessed by MTT assay after 24 h of treatment. Results are expressed as percentage of cell survival with respect to vehicle-treated cells. Data are mean ± SD of three independent experiments. ** *p* < 0.01 versus EGFR- cells; °° *p* < 0.01 versus the EGFR+ cells (one-way ANOVA followed by Tukey’s post hoc test). (**B**) EGFR+ were seeded at 30,000 cells/cm^2^ exposed to 100 µM of TMZ alone or in combination with 6 µM PF543 and/or 100 nM S1P. Cell viability was assessed by MTT assay after 24 h of treatment. Results are expressed as percentage of cell survival with respect to vehicle-treated cells. Data are mean ± SD of three independent experiments. *** *p* < 0.001 versus 100 µM TMZ, ** *p* < 0.01 versus control (one-way ANOVA followed by Tukey’s post hoc test). (**C**,**D**) EGFR+ cells were seeded at 30,000 cells/cm^2^ and grown in DMEM supplemented with 10% FCS were transfected with a mix of S57 and S59 siRNA for SK1 (siSK1) and the corresponding non-targeting mix of NTS59 as control (siCT) as described in Materials and Methods. At 72 h after transfection, (**C**) cell lysates (40 μg of protein) from two different preparations of siCT and siSK1 transfected cells were analyzed by immunoblotting with a polyclonal anti-SK1 antibody and monoclonal anti-GAPDH antibody; (**D**) cells were exposed to 100 µM of temozolomide. Cell viability was assessed by MTT assay after 24 h of treatment. Results are expressed as percentage of cell survival with respect to vehicle-treated cells. Data are mean ± SD of three independent experiments. * *p* < 0.05, ** *p* < 0.01, *** *p* < 0.001 versus siCT (*t*-test).

As shown in Figure 1A, the results demonstrated that the treatment with TMZ decreased cell viability in a concentration-dependent manner in EGFR- cells. Indeed, in EGFR- cells, the treatment with 100 μM TMZ was associated with a cell survival of 74%; meanwhile, that with 200 μM caused 65% of cell survival. Conversely, treatment with TMZ up to 200 μM did not cause any significant alteration in EGFR+ cells viability (Figure 1A). These data demonstrated that TMZ inhibits cell viability in EGFR- cells, but the expression of EGFRvIII protects the cells from the death effect of the drug and confers a TMZ-resistance phenotype.

### 2.2. S1P Produced by SK1 in EGFR+ Cells Confers Resistance to TMZ

An increasing amount of evidence demonstrates that S1P plays an important role as an oncopromoter lipid, involved in the mechanisms of resistance to chemotherapy in different tumors, including GBM [15,26,33]. Moreover, we demonstrated that the amount of extracellular S1P is significantly higher in EGFR+ cells vs. EGFR- cells due to the increased levels of the active form of SK1, the phosphorylated SK1 [31]. For this reason, we evaluated whether the expression of EGFRvIII, as well as the resistance to TMZ shown in Figure 1A, could be related to the increased production and release of S1P.

First, we evaluated the effect of the inhibition of SKs on TMZ-induced cytotoxicity by administering the drug together with a pan-SK inhibitor (SKI). We found that the co-treatment with TMZ and SKI (both at sub-toxic concentrations) significantly reduced EGFR+ cell viability (Figure 1A). In particular, in EGFR+ cells treated with 100 or 200 µM TMZ in the presence of a subtoxic dose of SKI, cell viability was decreased by 29% and 38%, respectively, compared to cells treated with TMZ alone (Figure 1A), obtaining a behavior very similar to the one we obtained with EGFR- cells treated with TMZ (Figure 1A). Of note, when a subtoxic dose of SK1 selective inhibitor PF543 was used, 100 µM TMZ significantly decreased (by 48%) EGFR+ cell survival (Figure 1B). Subsequently, in order to assess whether S1P is directly involved in EGFR+ acquired TMZ resistance, we evaluated the effect of nanomolar concentrations of S1P on cell survival in the presence of TMZ and PF543, separately or in combination. As shown in Figure 1B, in EGFR+ cells, the administration of nanomolar concentrations of S1P to control and TMZ-treated cells significantly increased the number of viable cells, supporting a growth- and survival-promoting effect of S1P (Figure 1B). Finally, and remarkably, the treatment with exogenous S1P resulted in the efficient reversion of the sensitizing PF543 effect on TMZ toxicity, determining a significant increase in viable cells (Figure 1B).

To further address the role of S1P, SK1-produced in EGFRvIII-induced survival, we determined whether the transient down regulation of SK1 by siRNA treatment in EGFR+ cells could affect the resistance of these cells to TMZ. To do this, we used Stealth RNAi, the chemically modified synthetic RNAi duplexes that virtually eliminate the induction of non-specific cellular stress response and that also improve the specific, effective knock-down of gene expression. First, we set up the optimal conditions to silence SK1. We used two Stealth RNAi (S57 and S59) targeting SK1 and the non-targeting corresponding sequences as control. After transfection with either S57 or S59, SK1 expression, as protein, was reduced by about 28% and 58% after 48 h and 72% and 88% after 72 h, respectively (Figure 1C). The greater efficiency of protein downregulation was achieved when cells were treated with S59 for 72 h (Figure 1C). In these experimental conditions, 100 µM TMZ significantly inhibited (by 28%) EGFR+ cell survival (Figure 1D). Taken together, these data indicate that the inhibition of SK1 increases the sensitivity of EGFR+ to TMZ toxicity, thus overcoming EGFR+ resistance to TMZ.

### 2.3. Extracellular S1P Is Responsible for the Resistance to TMZ of EGFR+ Cells

**Figure 2:**
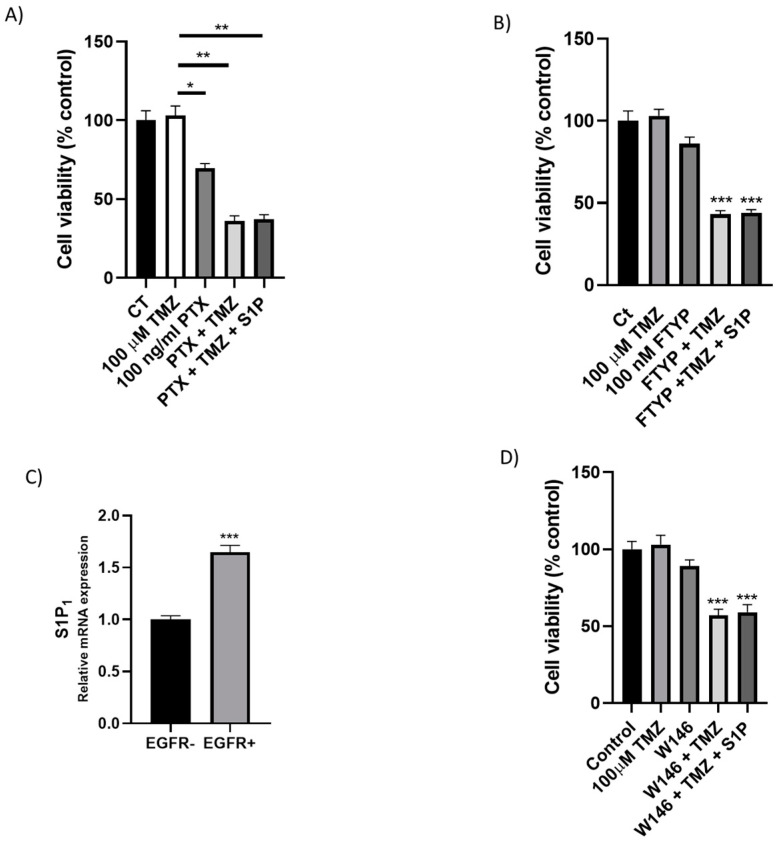
**Extracellular S1P promotes EGFR+ cell resistance to temozolomide.** EGFR+ cells were seeded at 30,000 cells/cm^2^ exposed to 100 µM TMZ alone or in combination with (**A**) 100 ng/mL PTX, (**B**) 100 nM FTY720. Cell viability was assessed by MTT assay after 24 h of treatment. Results are expressed as percentage of cell survival with respect to vehicle-treated cells. Data are mean ± SD of three independent experiments. (**C**) Cells were seeded and harvested as described in Materials and Methods. S1P_1_ mRNA expression by real-time PCR in EGFR- and EGFR+ cells. mRNA expression was normalized to the level of the housekeeping gene β-actin and referred to that of EGFR- cells. Data are shown as mean ± SD of independent experiments (*n* = 3). *** *p* < 0.001 versus control (*t*-test) (**D**) EGFR+ cells were seeded at 30,000 cells/cm^2^ exposed to 100 µM TMZ alone or in combination with 10 µM W146 with or without 100 nM S1P. Cell viability was assessed by MTT assay after 24 h of treatment. Results are expressed as percentage of cell survival with respect to vehicle-treated cells. Data are mean ± SD of three independent experiments. * *p* < 0.05, ** *p* < 0.01, *** *p* < 0.001 versus 100 µM TMZ (one-way ANOVA followed by Tukey’s post hoc test).

Similar to its effect in many other cell types [15,34], a low concentration of S1P enhanced cell viability in EGFR+ cells (Figure 2B). Since it has been demonstrated that SK1-generated S1P can act through S1P receptors stimulating cell proliferation and survival [19], we investigated the potential correlation between this “inside-out” signaling of S1P and EGFR+ cell survival. To this aim, by using heat-inactivated PTX, a blocker of Gi/Go–proteins [35], we demonstrated that PTX treatment decreased by about 30% basal EGFR+ cell viability (Figure 2A). Furthermore, we evaluated the effect of the inhibition of S1PRs on TMZ-induced cytotoxicity administering the drug together with the PTX. We found that the co-treatment with PTX and a subtoxic dose of TMZ significantly reduced EGFR+ cell viability (Figure 2A). In particular, in EGFR+ cells treated with 100 µM TMZ in the presence of PTX, cell viability was decreased by 64%, compared to cells treated with TMZ alone (Figure 2A), but the treatment with S1P did not affect the TMZ toxicity (Figure 2A). We also evaluated the effect of S-FTY720-v, the S1P_1–3–5_ receptors antagonist [36], on TMZ-induced cytotoxicity by administering the TMZ in combination with the S-FTY720v: the data we obtained demonstrated that cell viability was decreased by 57% in EGFR+ cells treated with 100 µM TMZ in the presence of S-FTY720v compared to cells treated with TMZ alone (Figure 2B); in addition, consistently with the results obtained with PTX, the treatment with exogenously administered S1P did not rescue TMZ-induced cell toxicity (Figure 2B).

Of note, the results obtained demonstrated that in EGFR+ cells, the mRNA level coding S1P_1_ was significantly higher than in EGFR- cells. In fact, in EGFR+ cells, S1P_1_ was 65% higher than the control cells (Figure 2C). The S1P_2–5_ receptors were not significantly expressed in both cell lines, EGFR- and EGFR+ cells (data not shown). On the basis of these results, we inhibited the receptor S1P_1_; when a subtoxic dose of the S1P_1_ specific inhibitor W146 was used, 100 µM TMZ significantly decreased (by 43%) EGFR+ cells survival (Figure 2D). Subsequently, to assess the effect of extracellular S1P in EGFR+ TMZ resistance, we evaluated the effect of nanomolar concentrations of exogenously administered S1P on cell survival in the presence of TMZ and W146, separately or in combination. The treatment with S1P did not revert the W146-induced sensitization to TMZ (Figure 2D).

### 2.4. Survival of EGFR+ Cells Requires ERK and AKT Activation Downstream of EGFRvIII

**Figure 3:**
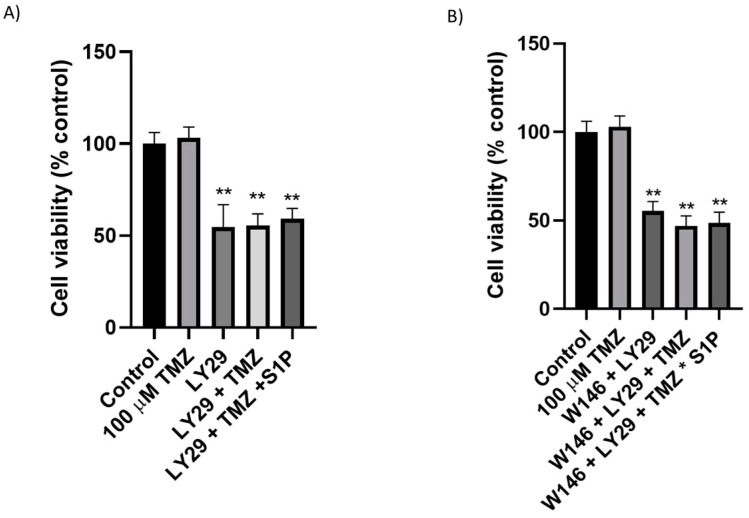
**Role of AKT on S1P-mediated survival of EGFR+ cells.** EGFR+ cells were seeded at 30,000 cells/cm^2^ exposed to 100 µM TMZ alone or in combination with (**A**) 20 µM LY294002, (**B**) 20 µM LY294002 together with 10 µM W146 with or without 100 nM S1P. Cell viability was assessed by MTT assay after 24 h of treatment. Results are expressed as percentage of cell survival with respect to vehicle-treated cells. Data are mean ± SD of three independent experiments. ** *p* < 0.01 versus control (one-way ANOVA followed by Tukey’s post hoc test).

We recently demonstrated that the phosphorylation of both ERK1/2 and AKT (at the Thr^202^/Tyr^204^ residues in ERK and at the Ser^473^ in AKT) were significantly higher in EGFR+ cells in comparison to EGFR- cells [31]. Furthermore, it is known that S1P and the PI3K/AKT pathways are involved in the regulation of cell proliferation/viability [37]. Therefore, we evaluated the involvement of the EGFRvIII-SK1-S1P-AKT axis in the survival properties of the EGFR+ cells. To this aim, we evaluated the effect of the inhibition of the signaling pathway PI3K/AKT on TMZ-induced cytotoxicity by the combined administration of the drug with the PI3K inhibitor LY294002. When LY294002 was used, EGFR+ cell viability was significantly reduced by 45%, and the co-treatment with 100 µM TMZ did not further inhibit (by 44%) EGFR+ cells survival (Figure 3A). The co-treatment with LY294002 and W146 did not further inhibit (by 45%) EGFR+ cell survival (Figure 3B), and the administration of 100 µM TMZ and of nanomolar concentrations of exogenous S1P together with TMZ did not significantly modify (by 53% and 51%, respectively) EGFR+ cell viability (Figure 3B).

**Figure 4:**
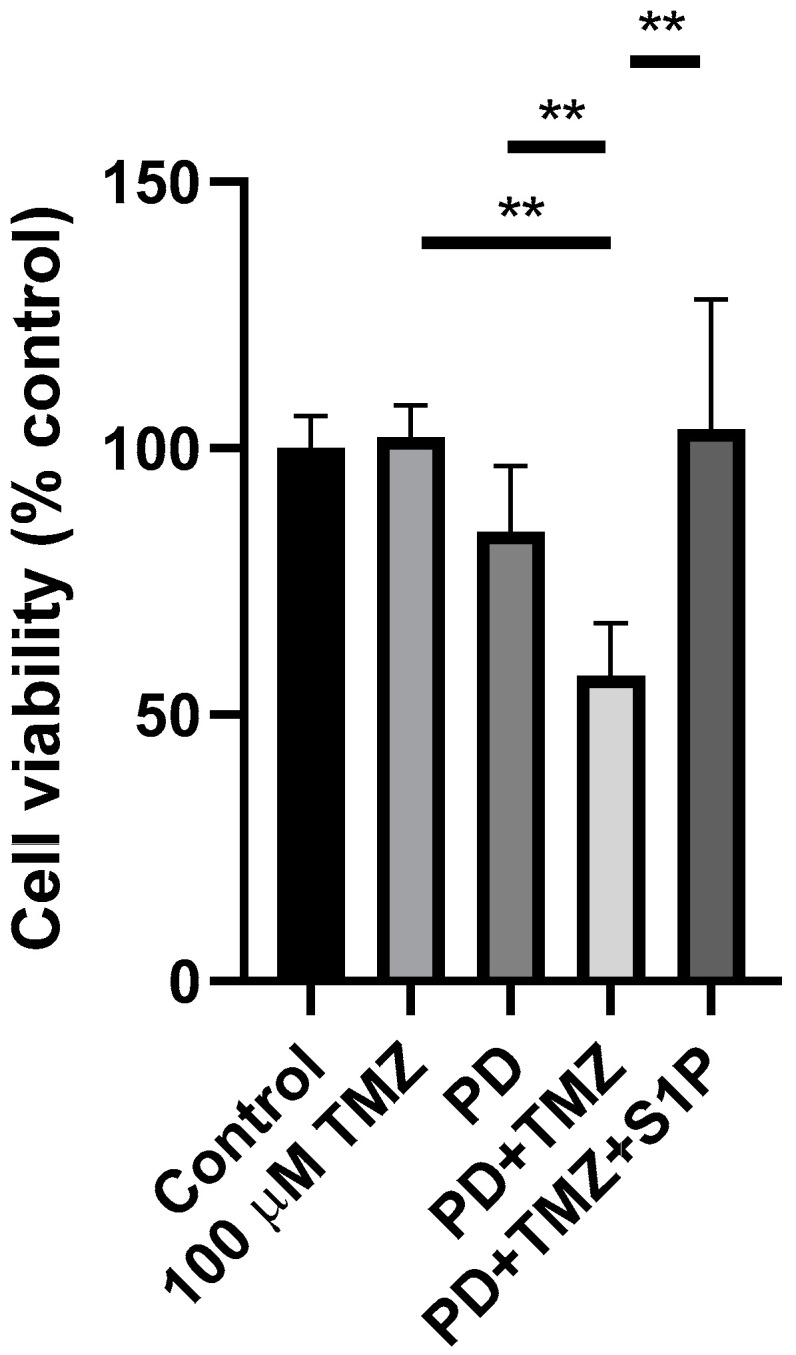
**Role of ERK on S1P-mediated survival of EGFR+ cells.** EGFR+ cells were seeded at 30,000 cells/cm^2^ exposed to 100 µM TMZ alone or in combination with 10 µM PD98059, with or without 100 nM S1P. Cell viability was assessed by MTT assay after 24 h of treatment. Results are expressed as percentage of cell survival with respect to vehicle-treated cells. Data are mean ± SD of three independent experiments. ** *p* < 0.01 (one-way ANOVA followed by Tukey’s post hoc test).

Subsequently, to assess the effect of extracellular S1P in EGFR+ LY294002-TMZ cell viability, we evaluated the effect of nanomolar concentrations of exogenously administered S1P on cell survival in the presence of TMZ and LY294002, separately or in combination; the treatment with S1P did not revert the effect of LY294002 on EGFR+ cell viability (Figure 3A). We published data demonstrating that in EGFR+, phosphorylated SK1 (pSK1) was increased and PD98059, the MEK1 inhibitor, significantly reduced pSK1 [31]; on these bases, we next evaluated the involvement of the EGFRvIII-ERK-SK1-S1P axis in the survival properties of the EGFR+ cells. We analyzed the effect of ERK inhibition on TMZ-induced cytotoxicity by administering the drug together with the ERK inhibitor PD98059. When a subtoxic dose of PD98059 was used, 100 µM TMZ significantly inhibited (by 43%) EGFR+ cells survival (Figure 4). We next evaluated the effect of nanomolar concentrations of exogenously administered S1P on cell survival in the presence of TMZ and PD98059; the treatment with S1P efficiently reversed the PD98059-TMZ induced toxicity, determining a significant increase in the number of viable cells (Figure 4).

Collectively, all these results suggest a role for the EGFRvIII and its downstream signaling pathways in the survival properties of S1P-mediated effects in the EGFR+ cells. Furthermore, these data indicate that S1P exerts its role in EGFR+ cell survival by its binding to and activation of S1P_1_.

## 3. Discussion

S1P is an oncopromoter lipid involved in the regulation of GBM growth and invasiveness [15,38,39,40], a tumor that, in turn, is characterized by an increase of the metabolic pathway that converts ceramide to S1P as well as by a decrease of the pathway that removes S1P for maintaining high levels of this last sphingoid molecule [41]. Moreover, it has been shown that SK1 is overexpressed in GBM, and this is directly associated with poor prognosis [16]. Several lines of evidence demonstrated that S1P stimulates the production and secretion of different growth factors, mainly EGF, PDGF, and VEGF [42]. Moreover, S1P has been shown to transactivate EGF/IGF receptor signaling pathways, resulting in increased GBM cell proliferation and tumor growth [43]. In particular, EGFR, in turn, hyperactivates the phosphatidylinositol 3′ kinase (PI3K)/AKT signaling pathway, which is involved in the regulation of glioma cell survival, proliferation and motility [44]. The constitutively active mutant form of EGFR, EGFRvIII, occurs frequently in GBM and confers a growth advantage to these tumors [44,45].

**Figure 5:**
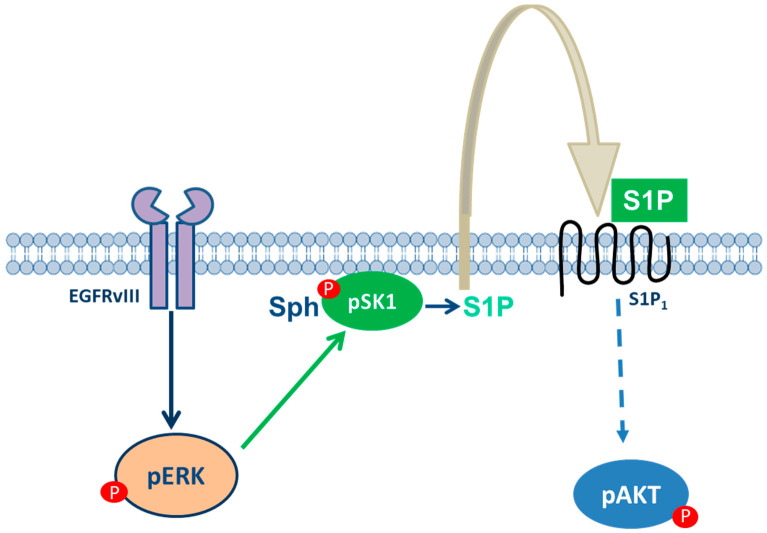
Scheme of the network of EGF-EGFR-SK1-S1P in human glioblastoma cells. In EGFR+ cells, phosphorylation of both ERK1/2 and AKT is significantly higher than in EGFR- cells. Moreover, in EGFR+ cells, pSK1, the activated form of SK1 is higher than in EGFR- cells, and it is dependent on ERK1/2 activation. SK1 activation leads to an increase in the extracellular S1P, which, in turn, binds to S1P_1_ receptors, thus promoting cell survival via AKT phosphorylation [31].

In GBM, the expression of the EGFRvIII variant has been correlated to the activation of SK1 [46] and to increased levels of extracellular S1P [31]; moreover, it has been shown that increased SK1 expression and S1P formation, transactivate EGFR signaling pathway [47]. We recently demonstrated in EGFR overexpressing glioma cells that extracellular S1P produced by SK1 is involved in the increased invasiveness through the activation of the EGFRvIII-ERK-SK1-S1P pathway via the S1P_1_ receptor [31] (Figure 5). Furthermore, GBMs are characterized by high resistance to the standard treatment with TMZ [48], suggesting that it is necessary to find and target different molecules able to amplify the effect of standard treatment. The prognosis for a patient affected by GBM is really poor; for this reason, the understanding of the mechanism able to drive chemoresistance is really important to develop possible therapies. High levels of growth factors are fundamental for GBM malignancy [49] as the EGFR signaling leads to downstream S1P production via SK1 activation [31,41].

In the present study, we demonstrate that human GBM cells stably overexpressing EGFRvIII are resistant to TMZ, the standard chemotherapeutic drug in GBM treatment, in spite of EGFR- cells. Our results also demonstrate that S1P, produced by the enzymatic action of SK1 and released in the extracellular milieu, acting through the S1P_1_ receptor is a survival signal in TMZ resistance shown by EGFRvIII expressing cells. Indeed, in EGFR+ cells resistant to TMZ, SK1 inhibition results in a decrease in cell viability following TMZ treatment; these results are consistent with previous data of literature, both in vitro and in vivo, demonstrating that SK1 inhibition leads to growth arrest of GBM cells [16,28,50,51] and when combined with TMZ treatment results in a reduction of cell viability [48,52]. To our knowledge, this is the first experimental evidence showing that the expression of EGFRvIII, through SK1 activation and the increased extracellular S1P levels via the S1P_1_ receptor, favours the survival of human GBM cells.

Our results demonstrate that cell viability is decreased by the pertussis toxin, which inactivates Gi/o type of G proteins; the S1P receptors are coupled to these proteins, and when S1P binds to the receptors, it activates a variety of downstream signaling pathways. Of note, in EGFR+ cells, the co-treatment with PTX and TMZ results in a significant decrease of cell viability. Furthermore, FTY720, a sphingosine analog that down-regulates the expression of S1P receptors, in combination with TMZ, synergistically induced a significant decrease of cell viability. Moreover, our results demonstrate that, in EGFR+ cells, S1P_1_ expression is significantly higher than in EGFR- cells and, in EGFR+ cells resistant to TMZ, the inhibition of the S1P_1_ receptor results in cell viability decrease after TMZ treatment according to data of literature demonstrating that S1P_1_ inhibition results in a cell viability decrease after TMZ treatment. Our results are in agreement with data published by Van Brocklyn demonstrating that S1P receptors inhibition with FTY720 results in growth arrest of GBM cells and the combination of FTY720 and TMZ synergistically induces apoptosis of GBM cells and slows the growth of intracranial xenograft tumors in nude mice increasing the therapeutic effect of TMZ, thus leading to enhanced survival [53]. On the other hand, a paper showed that S1P_1_ expression was much lower in glioblastoma than in normal brain [54] and, unexpectedly, that the downregulation of S1P_1_ expression enhanced the malignancy of GBM. The reason for this discrepancy between the results published by Yoshita et al. and the results published by Estrada-Bernal and Young, as well as our results demonstrating that S1P_1_ promotes glioma cell proliferation, is unclear [53,55]. All together, the results we obtained demonstrate that increase of SK1-produced S1P exerted its action through the S1P_1_ receptor and made the EGFR+ cells resistant to TMZ; in fact, the inhibition of SK1 or of S1P_1_ receptor made EGFR+ cells sensitive to TMZ. Indeed, the administration to the cells of exogenous S1P reverted the sensitization effect, thus involving extracellular S1P as a survival signal in TMZ resistance.

Several lines of evidence have been reported in the literature on the interaction between SK1-S1P and EGFR mediated signaling pathways. It has been demonstrated that S1P induces EGFR expression in vascular smooth muscle cells [30] or directly trans-activates EGFR in gastric cancer cells [56]. In lung adenocarcinomas, the S1P-S1P_3_ signaling upregulates EGFR and enhances EGFR-mediated carcinogenic activities contributing to tumorigenesis or progression of lung adenocarcinomas [29]. Moreover, in breast cancer cells, estrogens upregulate the S1P-SK1 pathway, and the produced S1P trans-activates EGFR via S1P_3_ [57]. Notably, it has been shown that S1P promotes the production and secretion of growth factors EGF, PDGF, and VEGF [42] and transactivates EGF/IGF receptor signaling pathways, promoting GBM cell proliferation and tumor growth [43].

Thus, the correlation between the pathways SK1-S1P and EGFR-mediated is very complex. We have recently demonstrated that, in GBM, EGFRvIII expression results in increased levels of phosphorylated AKT (pAKT) due to the PI3K pathway activation and SK1 inhibition exerted in decreased pAKT levels [31]. PI3K/AKT pathway inhibition reduced cell survival and TMZ did not further decrease cell viability. The administration of exogenous S1P did not restore cell viability, suggesting that the enhanced resistance to TMZ observed in the EGFR+ cells is dependent on the increased S1P secretion, downstream of EGFRvIII-SK1-S1P-PI3K/AKT pathway. Since the PI3K/AKT pathway is downstream of the SK1-S1P-S1P_1_ pathway, the administration of exogenous S1P is not able to bypass the PI3K/AKT pathway inhibition. Furthermore, EGFRvIII expression results in increased levels of phosphorylated ERK (pERK) [31], and our results demonstrated that inhibition of MAPK, which reduced pERK levels as well as phosphorylated SK1 [31], made the cells sensitive to TMZ, but the administration of exogenous S1P restores cell viability to the level of untreated cells.

These data suggest that, in GBM cells, the constitutively activated ERK pathway downstream of EGFRvIII in EGFR+ cells and responsible for SK1 phosphorylation and activation [31] is involved in the regulation of cell survival. In EGFR+ cells, MAPK, SK1 and S1P_1_ inhibitors made the cells sensitive to TMZ together with the ability of exogenously administered S1P to restore cell viability and resistance to TMZ, strongly indicating a crucial role of EGFRvIII and the downstream signaling ERK-SK1-S1P in the survival properties of EGFR+ GBM cells. Moreover, the data obtained demonstrate that S1P-mediated resistance in EGFR+ cells is mediated through S1P_1_ receptors; in fact, the selective chemical inhibition of S1P_1_ prevented the S1P-induced survival. Indeed, MAPK inhibitors reduced both SK1 activation and cell survival, suggesting that the enhanced resistance to TMZ observed in the EGFR+ cells is dependent on the increased S1P secretion, downstream of EGFRvIII-ERK-SK1-S1P pathway.

In conclusion, our data strongly indicate that, in GBMs, S1P production and release by EGRF+ cells downstream of the EGFRvIII-ERK-SK1-S1P_1_ pathway is involved in the pathogenic resistance to TMZ linked to EGFR overexpression and activation. All together, these results could partially explain the resistance of GBMs to treatments targeting EGFR [44]; in addition, our data further support the fundamental relevance of S1P signaling as a therapeutic target in these brain tumors. Thus, it is plausible that therapies targeting both S1P and EGFR signaling would be more effective strategies in the treatment of GBMs. Further studies using preclinical animal models are necessary to shed light on the potential clinical value of therapies directed against S1P- EGFR pathways to improve GBM outcome.

## 4. Materials and Methods

### 4.1. Materials

All reagents were of analytical grade. Dulbecco’s modified Eagle’s medium (DMEM), L-glutamine, penicillin, streptomycin, amphotericin B, fatty acid-free bovine serum albumin (FAF-BSA), 3-[4,5-dimethylthiazol-2-yl]2,5-diphenyl tetrazolium bromide (MTT), aprotinin, leupeptin, pepstatin, bestatin, EDTA, PD98059, LY294002, pertussis toxin (PTX), glycerol, β-mercaptoethanol, phenylmethylsulfonyl fluoride (PMSF), sodium glycerophosphate, NaF, Na_3_VO_4_, deoxypyridoxine, carboxymethylcellulose (M0512), and common chemicals were from Merck Life Science (Milan, Italy). Fetal calf serum (FCS) was from Euroclone (Pero, Milan, Italy). Sphingosine-1-phosphate (S1P) was from Enzo Life Sciences (Farmingdale, NY, USA). The antibodies anti-SK1, anti-GAPDH, and goat anti-rabbit or goat anti-mouse horseradish peroxidase-linked secondary antibodies were from Cell Signaling Technology, Inc. (Danvers, MA, USA). SuperSignal West Pico and West Femto Maximum Sensitivity Chemiluminescent Substrate were purchased from Pierce Chemical Co (Rockford, IL, USA). All solvents were purchased from Merck (Darmstadt, Germany). Sphingosine kinases inhibitor (SKI) was from Echelon Biosciences Inc. (Salt Lake City, UT, USA). PF543, CAY10444, and W146 and Temozolomide (TMZ) were from Cayman Chemical (Ann Arbor, MI, USA). S-FTY720-vinylphosphonate (S-FTY720-v) was kindly provided by Robert Bittmann, NewYork, USA. RNeasy Mini kit was provided by Qiagen (Hilden, Germany), iScript cDNA Synthesis kit, SYBR Green (iQ SYBR Green Supermix were from Bio-Rad (Hercules, CA, USA) Lipofectamine 2000, siRNA and primers for real-time PCR were from Invitrogen (Waltham, MA, USA).

### 4.2. Cell Cultures

U87MG human GBM cell line overexpressing EGF receptor variant III (EGFvIII) (EGFR+ cells) (kindly provided by Prof. Pier-Luigi Lollini, University of Bologna, Italy) or overexpressing empty vector (EGFR-) were cultured in DMEM containing 10% (*v*/*v*) FCS, 2 mM L-glutamine, 100 units/mL penicillin, 100 μg/mL streptomycin, and 0.25 μg/mL amphotericin B at 37 °C in 5% CO_2_ humidified atmosphere. Stable transfectants of EGFR- and EGFR+ cells were maintained in medium containing 1 g/L G418.

### 4.3. Cell Treatments

For cell treatments, stock solutions were prepared by dissolving the different molecules as follows: TMZ, SKI, PF543, W146, CAY10444, LY294002 and PD98059 in DMSO; S1P and S-FTY720-v in 4 mg/mL fatty acid-free bovine serum albumin (FAF-BSA) in PBS; PTX in H_2_O. Stock solutions were diluted extemporaneously in fresh medium at the desired concentrations and administered to cells in the absence of Amphotericin B for the indicated periods of time. In each experiment, cells were also incubated with vehicles as controls.

### 4.4. Analysis of Cell Viability

Cell viability was determined by MTT assay. EGFR- and EGFR+ cells were seeded at 1 × 10^4^ and 2 × 10^4^ cells/cm^2^, respectively, in 96-well plates. The day after, cells were treated with different agents for the indicated periods of time. The medium was then replaced by MTT dissolved in fresh medium (0.8 mg/mL) for 4 h. The formazan crystals were then solubilized in iso-propanol/formic acid (95:5 *v*/*v*) for 10 min and the absorbance (570 nm) was measured using a microplate reader (Wallack Multilabel Counter, Perkin Elmer, Boston, MA, USA).

### 4.5. RNA Interference

Small interfering RNA (siRNA) duplexes for human SK1 5′-UCACGCUGAUGCUCACUGA-3′, (S57), 5′-AACUACUUCUGGAUGGUCATT-3′, (S59), and control siRNAs, non-targeting, scrambled sequences of S57 and S59 oligonucleotide (NTS57; NTS59) were selected using the Invitrogen RNAi Designer software and obtained from Invitrogen. The lack of targeting for other genes expressed by U87-MG by the designed siRNA duplexes was then checked by BLAST to avoid silencing of multiple genes other than SK1. EGFR+ glioma cells plated at 3.0 × 10^4^ cells/cm^2^ were maintained 24 h in DMEM plus 10% FCS and then transfected in the same medium with 100 nM (final concentration) of the following oligonucleotides: S57, S59 and the non-targeting corresponding sequences, using Lipofectamine 2000, according to the manufacturer’s protocol. Specific silencing achieved 48 and 72 h after transfection was evaluated by immunoblotting. All the experiments were done with the two different 21-nucleotide duplexes and to rule out off-target effects, and the data shown were obtained after treatment of the cells with S59.

### 4.6. Immunoblotting Analysis

Cells were lysed with lysis buffer (20 mM Tris-HCl, pH 7.4, 150 mM NaCl, 1% Nonidet P-40, 10 mM NaF, 1 mM EDTA, 10 mM Na_4_P_2_O_7_, 1 mM Na_3_VO_4_, and the protease inhibitor mixture).

In order to evaluate SK1 and GAPDH expression, cell proteins were resolved by SDS-PAGE on 10% polyacrylamide gels and transferred onto PVDF membranes. Membranes were then blocked with 3% BSA in TBS with 0.05%-Tween-20, incubated overnight with anti-SK1 (1:1000) and anti-GAPDH (1:2000) primary antibodies and finally with goat anti-rabbit or anti-mouse horseradish peroxidase-linked secondary antibodies (1:3000 or 1:4000, respectively) using GAPDH as the loading control.

In all cases, bound antibodies were visualized by ECL (SuperSignal West Pico or West Femto Maximum Sensitivity Chemiluminescent Substrate). For quantitative measurements, immunocomplexes were visualized by UVITEC Cambridge technology (Eppendorf). Image analysis was performed using NINEAlliance software.

### 4.7. Real-Time RT-PCR

RNA (800 ng) was purified employing the RNeasy Mini kit and further retro transcribed into cDNA using the iScript cDNA Synthesis kit. Expression of S1P receptors was evaluated through real-time PCR. PCR reaction mixture (20 µL) was constituted of cDNA (corresponding to 10 ng of total RNA), 0.2 mM primers, 50 mM KCl, 20 mM Tris/HCl pH 8.4, 0.8 mM dNTPs, 0.7 U iTaq DNA Polymerase, 3 mM MgCl_2_, and SYBR Green (iQ SYBR Green Supermix). Amplification was performed using the iCycler iQ5 thermal cycler (Bio-Rad), as follows: initial denaturation at 95 °C for 3 min, 45 cycles of 10 s at 95 °C, and 30 s at 59 °C. The fold change in expression of genes encoding S1P receptors was normalized to β actin expression and evaluated by the equation 2^−∆∆Ct^, comparing EGFR+ cells to EGFR- cells. At the end of amplification, melting curves were performed. The following primers were used: β actin, Forward: 5′-CGACAGGATGCAGAAGGAG-3′; Reverse: 5′-ACATCTGCTGGAAGGTGGA-3′; S1P_1_, Forward: 5′-AAATTCCACCGACCCATGTA-3′; Reverse: 5′-AGTTATTGCTCCCGTTGTGG-3′; S1P_2_, Forward: 5′-CACCTGGCGGTACAAAGAAT-3′; Reverse: 5′-GTCAAGTGGCAGCTGATGAA-3′; S1P_3_, Forward: 5′-GCTTCAGGAAATGGAAGCTG-3′; Reverse: 5′-TCAGGATGCTGTGAAACTGC-3′; S1P_4_, Forward: 5′-CTGCTCTTCACCGCCCTGGC-3′; Reverse: 5′-GAAGCCGTAGACGCGGCTGG-3′; S1P_5_, Forward: 5′-GTGAGGTGGGAGCCATAGAA-3′; Reverse: 5′-TTGGCTGAGTCTCCCAGAGT-3′

### 4.8. Statistical Analysis

Results are expressed as means ± SD for at least three independent experiments. The statistical significance of the data was determined by the Student’s *t*-test or by the one-way analysis of variance (ANOVA) followed by post hoc Tukey test, when applicable. Significant differences at least *p* < 0.05 were accepted.

## Data Availability

The original image of the western blot presented in this study is available on request from the corresponding author.
